# Ocean acidification reverses the positive effects of seawater pH fluctuations on growth and photosynthesis of the habitat-forming kelp, *Ecklonia radiata*

**DOI:** 10.1038/srep26036

**Published:** 2016-05-27

**Authors:** Damon Britton, Christopher E. Cornwall, Andrew T. Revill, Catriona L. Hurd, Craig R. Johnson

**Affiliations:** 1 Institute for Marine and Antarctic Studies, University of Tasmania, Hobart, TAS 7001, Australia; 2 School of Earth and Environment, Oceans Institute, & ARC Centre of Excellence for Coral Reef Studies, University of Western Australia, Crawley, WA 6009, Australia; 3 CSIRO Oceans and Atmosphere, Hobart, TAS, Australia

## Abstract

Ocean acidification (OA) is the reduction in seawater pH due to the absorption of human-released CO_2_ by the world’s oceans. The average surface oceanic pH is predicted to decline by 0.4 units by 2100. However, kelp metabolically modifies seawater pH via photosynthesis and respiration in some temperate coastal systems, resulting in daily pH fluctuations of up to ±0.45 units. It is unknown how these fluctuations in pH influence the growth and physiology of the kelp, or how this might change with OA. In laboratory experiments that mimicked the most extreme pH fluctuations measured within beds of the canopy-forming kelp *Ecklonia radiata* in Tasmania, the growth and photosynthetic rates of juvenile *E. radiata* were greater under fluctuating pH (8.4 in the day, 7.8 at night) than in static pH treatments (8.4, 8.1, 7.8). However, pH fluctuations had no effect on growth rates and a negative effect on photosynthesis when the mean pH of each treatment was reduced by 0.3 units. Currently, pH fluctuations have a positive effect on *E. radiata* but this effect could be reversed in the future under OA, which is likely to impact the future ecological dynamics and productivity of habitats dominated by *E. radiata*.

Ocean acidification (OA) is the decline in surface seawater pH caused by the sustained absorption of anthropogenically-derived atmospheric CO_2_^1^. OA is predicted to cause widespread change in many marine ecosystems^2^,^3^, primarily through its potential to reduce the net calcification rates of calcareous species, and to alter the behaviour of invertebrates and fishes^4^,^5^. However, some species of non-calcareous macroalgae may benefit from OA^6^,^7^, including canopy-forming kelps^8^,^9^.

Canopy-forming kelps are species of large, brown macroalgae of the orders Laminariales (true kelps) and Fucales (functional equivalents) that form extensive forests or beds between latitudes of ~35–65° 10,11. Kelps play a pivotal role in providing habitat, food, and nursery areas for numerous species, and they strongly influence the structure of understorey invertebrate and macroalgal assemblages, primarily through the alteration of light and water motion^12^,^13^,^14^. The majority of research into the resilience and stability of kelp forests has been concerned with external processes such as over-fishing, increased temperature, or terrestrial inputs that act to disturb or maintain the system^15^,^16^,^17^,^18^,^19^. However, internal processes may also be important in maintaining kelp forests through feedback mechanisms^18^,^20^,^21^. One internal mechanism could be the capacity of kelp to elevate seawater pH via their photosynthetic activity^22^,^23^,^24^. The diel range in seawater pH within some kelp beds can be almost 1 pH unit (i.e. ±0.45) due to the uptake of dissolved inorganic carbon (DIC) via photosynthesis during the day that causes pH to increase, and respiration which produces CO_2_ during the night, thus lowering seawater pH^22^,^25^. The largest daily pH fluctuations of 0.90 units (7.96–8.86) have been recorded in giant kelp forests (*Macrocystis pyrifera*)^22^,^23^, leading to suggestions that this process may buffer these systems from the effects of OA^22^,^26^,^27^,^28^,^29^.

It is unclear how OA will influence kelp physiology and ecology^6^,^30^,^31^, and it is unknown how kelps respond to fluctuations in pH caused by their own metabolic activity. For non-calcareous (i.e. fleshy and foliose) macroalgae, the effects of OA are variable, increasing the growth and photosynthetic rates of certain species^6^,^9^,^32^, decreasing the growth rates of others^33^,^34^,^35^, or having no detectable effect^30^,^31^,^36^. No study to date has examined the response of non-calcareous macroalgae to fluctuations in pH (although the recruitment of a range of fleshy macroalgae was reported in a study examining the effects of pH fluctuations on juvenile coralline algae^37^). For coralline algae, pH fluctuations negatively affect growth rates, under both ambient mean pH levels and under mean levels expected for the future as a result of OA^22^,^37^,^38^. For kelp, however, reductions in seawater pH may have little effect on photosynthesis and growth, because they actively uptake HCO_3_^−^ (the most abundant form of DIC in seawater) using carbon concentrating mechanisms (CCMs)^8^,^31^,^39^. Alternatively, the projected 200% increase in seawater CO_2_ concentration could benefit kelp by increasing the diffusive uptake of CO_2_, which might result in the down-regulation of the CCM^8^. Increased uptake of CO_2_ could therefore result in elevated rates of growth and photosynthesis for kelp^8^. However, in kelp beds the high (metabolically-induced) daytime pH might have an opposing effect because CO_2_ concentrations are reduced as pH increases, and so in this case growth and photosynthetic rates might be reduced.

*Ecklonia radiata* is a dominant canopy-forming member of the order Laminariales in the Southern Hemisphere, ranging from South Africa, to southern Australia and New Zealand^10^. Despite its importance as an ecosystem engineer, creating habitat and food for thousands of species^40^, we know almost nothing about the pH environment that it experiences daily, nor its responses to pH fluctuations, both now and those predicted to occur in the future. To address this knowledge gap, we took measurements of pH within several *E. radiata* beds in south eastern Tasmania, Australia, to assess the daily pH fluctuations. Using these field values to provide an environmental context for laboratory experiments, in one experiment we grew juvenile *E. radiata* under fluctuating (8.1 + 0.3 units in the day and −0.3 units in the night) and constant pH regimes (pH 8.4, 8.1, and 7.8) over 21 days in the laboratory, and in a second similar experiment at pH −0.3 units in all treatments (hereafter “ambient” and “OA” respectively). We hypothesized that: 1) Seawater pH fluctuates on a diel cycle within *E. radiata* beds due to their photosynthetic activity, reducing at night and increasing during the day; 2) simulated fluctuations in pH will reduce growth (measured as a change in thallus length) and photosynthetic rates (O_2_ evolution) of *E. radiata* compared to static pH treatments with the same mean pH possibly as a result of up-regulation of the CCM; 3) pH conditions simulating OA (0.3 pH_NBS_ units lower than present day) will cause an increase in *E. radiata* growth and photosynthetic rates relative to treatments with present day (8.1) and high pH (8.4); and 4) RNA:DNA ratios will increase in treatments where growth increases, reflecting increased protein synthesis and hence increased total RNA content. The carbon isotope composition of seaweed tissue can be used to infer changes in the relative use of CO_2_ and HCO_3_^−^ 22,31,36, because CO_2_ is more depleted in ^13^C (i.e. its δ^13^C is lower) compared to HCO_3_^−^ 41. Thus, we also hypothesized that 5) *E. radiata* Δ^13^C (i.e. the difference between tissue and source seawater DIC in each treatment) would increase with declining pH (i.e. increasing CO_2_) as a result of increased use of diffusive CO_2_ over active uptake of HCO_3_^−^ 42.

## Results

### Field Measurements

Seawater pH showed clear diel cycles at all 3 sites where pH was measured. The range in pH on the total scale (pH_T_; all subsequent field measurements are referred to on the total scale) was larger (0.40 units) within the more sheltered, shallower sites (where pH was measured only during daylight hours over 3 days) than at deeper, more wave-exposed sites (0.05–0.09 units; over 21 days). Seawater pH within the sheltered, shallow (1.5 m depth) *E. radiata*/*Phyllospora comosa* bed at Darlington, Maria Island, displayed a clear increase over the course of the day, with a minimum of pH 7.97 ± 0.06 at 08:00 on day 2 and maximum of 8.37 ± 0.01 at 14:00 on day 3 (Table 1). pH was tightly correlated with oxygen concentration (*r* = 0.89 ± 0.06, *P* < *0.001*). Seawater pH in Fortescue Bay in late autumn and mid-winter showed a similar diel pattern, but it had a smaller range than at Maria Island; pH fluctuated 0.08 pH units (8.01 to 8.09) at 12.5 m depth over 3 days. At the mouth of Fortescue Bay in early spring, pH varied by 0.09 pH units (8.06 to 8.15) over 21 days at 7 m, and 0.05 units (8.06 to 8.11) over 21 days at 25 m (Table 1). At this site pH was tightly correlated with oxygen concentration at 7 m (*r* = 0.93, *P* < 0.01), but only weakly correlated with oxygen concentration at 25 m (*r* = 0.37, *P* < 0.01). pH within the *E. radiata* canopy, determined from Niskin sampling in late spring, ranged from 8.13 to 8.19 over daylight hours with the lowest values occurring at 06:45 and the highest values occurring at 16:45, while pH measurements made over the same time period above the adjacent soft sediment benthos ranged between 8.15 and 8.16 pH units with no indication of diel fluctuations.

pH within sealed bags containing adult *E. radiata* sporophytes in the shallow *E. radiata*/*Phyllospora comosa* bed increased significantly more (from 8.00 ± 0.02 to 8.72 ± 0.05) over the course of the day than control bags without *E. radiata* (from 8.00 ± 0.02 to 8.14 ± 0.03; ANOVA, *F*_2,17_ = 142.31, *P* = <0.01). Oxygen concentrations also increased significantly more (84.43%) within the bags containing *E. radiata* compared to controls (ANOVA, *F*_1,17_ = 36.86, *P* < 0.01). Oxygen concentration was positively correlated with pH, both in bags with and without *E. radiata* (*r* = 0.94 ± 0.02).

### Laboratory experimental conditions

pH treatments within both of the experiments were maintained within 0.05 units of the treatment target throughout all experiments, and the standard error of pH in each tank for each treatment was smaller than the measurement error (<0.01). The pH_T_ of treatments (with treatment pH_NBS_ labels in parentheses) was 7.78 (7.8), 8.09 (8.1) and 8.35 (8.4) for growth experiment 1 and 7.54 (7.5), 7.80 (7.8) and 8.06 (8.1) for growth experiment 2. Alkalinity (A_T_) did not vary substantially across treatments within experiments, being 2294.57 ± 10.79 μmol kg^−1^(mean ± s.e.) in the first experiment and 2352.66 ± 16.40 μmol kg^−1^ in the second. DIC measured in each treatment was inversely related to the pH of that treatment (Table S1). Salinity was 35.00 ± 0.01 in each treatment.

### Growth experiment 1: ambient seawater, mean pH 8.1

**Figure 1:**
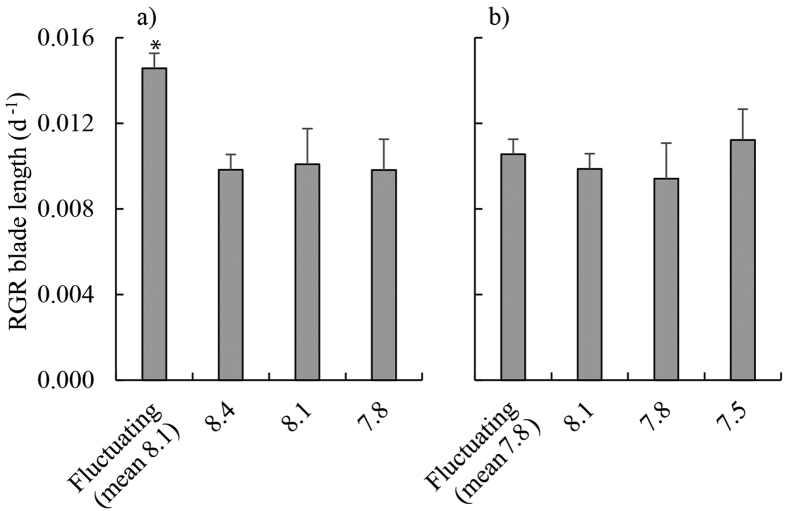
Relative growth rates (RGR) of juvenile *Ecklonia radiata* over 21 days, measured as linear extension of the blade under (**a**) ambient pH conditions (fluctuating pH_NBS_ [8.4 during the day, 7.8 at night], constant pH_NBS_ at 8.4, 8.1 and 7.8); and (**b**) OA conditions (the same treatments as in (**a**) but with pH_NBS_ reduced by 0.3 units in each treatment). Data are displayed as means ± standard error, *n* = 4–6. * denotes significantly different treatments, as revealed by Tukey’s Honestly Significant Difference tests (α = 0.05).

**Figure 2:**
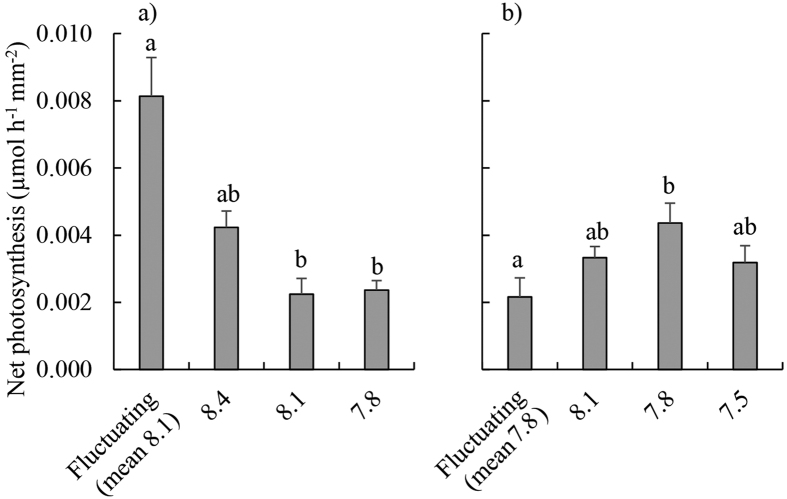
Net photosynthetic rates of juvenile *Ecklonia radiata* (μmol O_2_ h^−1^ mm^−2^) after 21 days under treatments of: ambient pH conditions (fluctuating pH_NBS_ [8.4 during the day, 7.8 at night], constant pH_NBS_ at 8.4, 8.1 and 7.8); and (**b**) OA conditions (the same treatments as in (**a**) but with pH_NBS_ reduced by 0.3 units in each treatment). Data are displayed as means ± standard error, *n* = 4–6. Bars sharing a letter within panels are not significantly different, as revealed by Tukey’s Honestly Significant Difference tests (α = 0.05).

**Figure 3:**
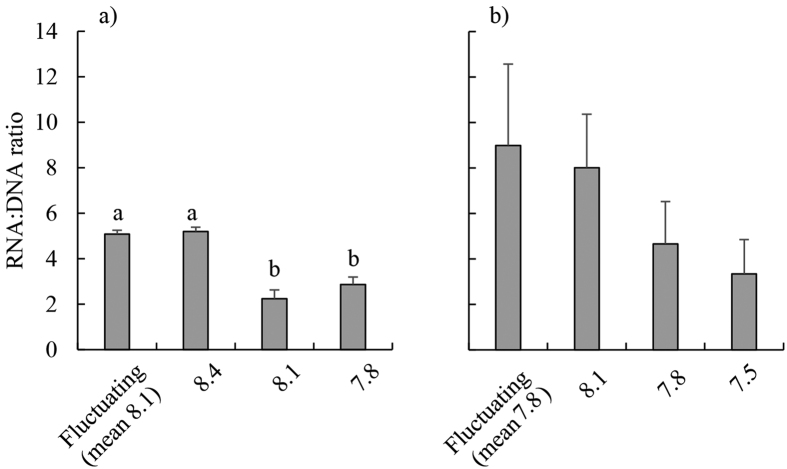
RNA:DNA ratios of juvenile *Ecklonia radiata* after 21 days under the following treatments: (**a**) ambient pH conditions (fluctuating pH_NBS_ [8.4 during the day, 7.8 at night], constant pH_NBS_ at 8.4, 8.1 and 7.8); and (**b**) OA conditions (the same treatments as in (**a**) but with pH_NBS_ reduced by 0.3 units in each treatment). Data are displayed as means ± standard error, *n* = 4–6. Bars sharing a letter within panels are not significantly different, as revealed by Tukey’s Honestly Significant Difference tests (α = 0.05).

**Figure 4:**
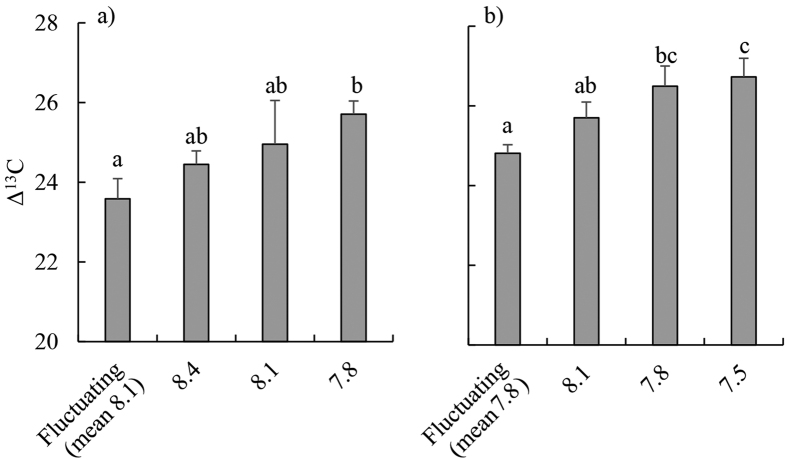
Δ^13^C isotope ratios (tissue δ^13^C corrected for source dissolved inorganic carbon δ^13^C) of juvenile *Ecklonia radiata* after 21 days grown under: (**a**) ambient pH conditions (fluctuating pH_NBS_ [8.4 during the day, 7.8 at night], constant pH_NBS_ at 8.4, 8.1 and 7.8); and (**b**) OA conditions (the same treatments as in (**a**) but with pH_NBS_ reduced by 0.3 units in each treatment). Data are displayed as means ± standard error, *n* = 3–6. Bars within panels sharing a letter are not significantly different, as revealed by Tukey’s Honestly Significant Difference tests (α = 0.05).

Relative growth rates of blades were on average 47% greater in the fluctuating pH treatment than in all other treatments (Table 2, Fig. 1a). Net photosynthetic rates were higher (on average 253% higher) in the fluctuating pH treatment compared to the static pH 7.8 and pH 8.1 treatments (Table 2, Fig. 2a). RNA:DNA ratios were significantly higher in the fluctuating and static pH 8.4 treatments than at constant pH 8.1 and pH 7.8 (mean 101% higher, Tukey’s Honestly Significant Difference (THSD), *P* < 0.01 for all significant differences) Fig. 3a) and this was caused by RNA content being 198% higher in these treatments (Table 2, Fig. S1a). There was no significant effect of pH treatments on DNA content (Table 2, Fig. S1c). There was no effect of treatment on rETR_max_ (Table 2, Fig. S4a). F_v_/F_m_ was highest at pH 8.4 (0.73 ± 0.01) and lowest at pH 8.1 (0.69 ± 0.01; Table 2, THSD: *P* = 0.015, Fig. S2a), but at all times was within the range indicating that photosystem II (PSII) was functioning normally. Δ^13^C values of individuals increased as the day time pH of treatments decreased (Fig. 4a). Δ^13^C values were significantly higher in the fluctuating treatment compared to the static pH 7.8 treatment (Table 2). C:N ratios were significantly higher in the static pH 7.8 treatment than in all other treatments (Table 2, Fig. S3a).

### Growth experiment 2: OA conditions, mean pH 7.8

Relative growth rates were similar between treatments (Table 2, Fig. 1b). However, net photosynthesis was 49% lower in the fluctuating pH treatment relative to the static pH 7.8 treatment (Table 2, THSD: *P* = 0.03, Fig. 2b). There were no significant differences between treatments in RNA:DNA ratios (Table 2, Fig. 3b), total RNA, and total DNA content (Table 2, Fig. S1). There was no effect of experimental treatment on rETR_max_ (Table 2, Fig. S4b). Although F_v_/F_m_ was significantly lower in the fluctuating pH treatment (0.69 ± 0.01, mean ± s.e.) than the pH 7.8 (0.72 ± 0.01) and 8.1 (0.73 ± 0.01) treatments (Table 2, Fig. S2b), again these values indicate normal functionality of PSII. Similar to Experiment 1, Δ^13^C values increased with decreasing pH during the day, and were significantly higher in the pH 7.5 and 7.8 treatments compared to the fluctuating pH treatment (Table 2, TSHD: *P* = 0.02 and 0.03, Fig. 4b). C:N was similar for all treatments (Table 2, Fig. S3).

## Discussion

We measured diel changes in pH within *E. radiata* beds of up to 0.4 units, and found that when these fluctuations are simulated in laboratory experiments, rates of blade growth and photosynthesis (measured as O_2_ evolution) of juvenile *E. radiata* increased compared to static pH treatments. However, this positive effect of fluctuating pH on growth and photosynthetic rates was not apparent when the experimental pH was reduced in all treatments by 0.3 units, simulating future OA. Moreover, pH fluctuations under simulated OA had a negative impact on net photosynthesis relative to static pH treatments with the same mean pH.

These findings are important in the context of predicting the responses of kelp-based communities to OA. Past research has considered that metabolically-induced pH fluctuations in habitats dominated by photosynthetic species (macroalgae, seagrasses, corals) could act as a refuge from OA for calcifying species because higher pH during the day might facilitate calcification^22^,^26^,^43^,^44^. However, this refuge may be less effective than previously considered in a future, reduced pH ocean, if these pH fluctuations no longer benefit the kelp that are responsible for the pH changes. Increased variability in pH has a negative impact on coralline algae under ambient mean seawater pH, the effects of which are enhanced by OA^22^,^37^. However, in the case of coralline algae, reduced pH in general is known to slow growth and net calcification, possibly through elevated dissolution rates^22^,^43^,^45^. The mechanisms responsible for the negative impacts (relative to fluctuating pH under current conditions) of fluctuating pH under OA conditions on the non-calcareous *E. radiata* are unclear (see below). Further research is required to determine whether other wide-spread and abundant habitat-forming macroalgal species will respond to pH fluctuations predicted for the future in a similar way to *E. radiata*, or whether this is a species-specific response.

pH fluctuations were not spatially or temporally uniform within the *Ecklonia radiata* beds, further indicating that the ability of habitats dominated by photosynthetic species to act as a refuge from OA is context-dependent. The largest range in pH observed over 24 hours (0.40 pH units) was at the shallowest and most wave-sheltered site (Maria Island), and the smallest range in pH (0.05 pH units) was at the outermost site at Fortescue Bay at 25 m depth which is both the most wave-exposed and deepest site. It was not our objective to determine the environmental factors responsible for different pH ranges within *E. radiata* beds, nor do the data allow us to do so, however the consistent trends of pH fluctuations we observed support findings that the extent of mixing and water retention within a habitat and depth^44^,^46^,^47^ influence the magnitude of pH fluctuations in macrophyte systems. While we used several methods to measure pH of the seawater *in situ*, they each followed the recommended best practice^48^ and we do not consider that differences between methods influenced the trends observed. Before effective predictions can be made regarding the extent of pH change likely to occur in various macrophyte-dominated habitats, further research is required that combines physical modelling with long-term, spatially-extensive measurements of pH, water motion, depth and biotic characteristics that are replicated through space and time. Once these data are obtained, the role that macrophyte habitats could play in modifying the effects of OA on resident organisms can be more thoroughly understood.

Contrary to our initial hypotheses, the net photosynthetic and growth rates of *Ecklonia radiata* were not elevated in treatments with reduced daytime pH (and more CO_2_) relative to present ocean conditions. This supports past research indicating that increased concentrations of DIC, particularly CO_2_, associated with OA may not benefit this species^6^. The Δ^13^C of *E. radiata* displayed a clear trend of a greater reliance on diffusive CO_2_ as a carbon source for photosynthesis in treatments with lower daytime pH, as hypothesized initially. However, we saw no evidence of increased growth rates as might be expected if CCMs are down-regulated, despite the increasing reliance on diffusive CO_2_ at low pH; this is similar to the growth responses of *Ulva rigida* and *Macrocystis pyrifera*^31^,^36^ to elevated CO_2_.

Rather than increased growth at higher DIC concentrations (i.e. lower pH), we found that the rates of *E. radiata* growth and photosynthesis were maximal under the experimental treatment that most closely simulated the pH fluctuations that it currently encounters in the field (daytime pH 8.4, night-time pH 7.8), suggesting that it is physiologically adapted to these pH/DIC conditions. We suggest that the specific combination of a daytime pH 8.4 and night-time pH 7.8 provides a seawater carbonate chemistry that is conducive to growth, photosynthesis, and RNA synthesis by *Ecklonia*. The static pH 8.4 treatment itself appeared to be slightly beneficial to *Ecklonia* because RNA synthesis was increased, along with evidence of increased O_2_ evolution, although there was no evidence of increased growth. The explanation for this finding of stimulated metabolism when the daytime pH was 8.4 may be related to enhanced bicarbonate uptake at higher pH, which has been previously observed at very high pH (~9.0) for green seaweeds, but not brown seaweeds^49^. However, we know relatively little of the diversity of bicarbonate uptake mechanisms in kelps, although this knowledge is required to further understand how they will respond to OA (see references within^31^,^50^).

This is the first documentation of seaweed metabolism being enhanced by diel fluctuations in seawater carbonate chemistry. The mechanisms are unknown but are likely to involve metabolic processes that are enhanced by higher H^+^ and/or DIC at night, along with decreased H^+^ and/or DIC during the day. Further studies are needed including those using enzyme inhibitors and gene expression, to elucidate why this specific combination of pH/DIC during the day and night stimulated growth of *E. radiata*. The next step in quantifying how seawater carbonate chemistry influences growth and photosynthesis of macroalgae should investigate the activity of different CCMs in macroalgae grown for long periods of time under pH treatments similar to ours. This can be done by combining direct assessments of specific CCMs^50^ with gene expression data.

This study is the first to examine the effects of fluctuations in pH on a non-calcareous macroalgae, and our findings build on those of earlier studies^22^,^37^,^38^ which highlight the importance of acknowledging pH fluctuations when assessing how ecologically important calcifying and non-calcifying primary producers might be affected by ocean acidification in dynamic coastal environments. The finding that the positive effects of fluctuating pH were not apparent under OA has implications for the abilities of kelps to act as refugia for calcifying organisms in the future (cf 27,28,43). This study raises many questions, but it now seems clear that it is important to ascertain how the magnitude of macrophyte-induced diel pH fluctuations in shallow environments influence the physiology and ecology of marine species now and in a future lower pH ocean. How different magnitudes of fluctuations combine with changes in mean pH to influence organisms now needs to be addressed for a range of species before we can begin to predict how long-term changes in near-shore pH due to OA could impact these ecosystems.

## Methods

### Field measurements

To determine whether pH within *Ecklonia radiata* beds follows a daily cycle similar to those occurring in other ecosystems^22^,^24^, seawater pH (and where possible dissolved oxygen) was measured on five different sampling occasions at two general locations, *viz*. Darlington (Maria Island), and Fortescue Bay, Tasmania:

(1) In a shallow (1.5 m), sheltered *E. radiata/Phyllospora comosa* bed, an environment likely to have extreme pH fluctuations (Darlington), at regular intervals between 07:30 and 17:30 on each day between April 19-21 2014. At this location pH and dissolved oxygen (DO) were measured using a pH meter (Thermo Scientific Orion Star A216 pH/RDO/DO meter), pH electrode (Thermo Scientific Orion 8107 BNUMD Ross Ultra pH/ATC Triode) and DO probe (Thermo Scientific Orion 087100MD Field RDO probe). Seawater was collected *in situ* using bottles (100 ml plastic sealed bottles) because surge prohibited the use of sensors in the shallow water;

(2) At 7 m and 25 m depth on an exposed coast (Fortescue Bay) where pH fluctuations are likely to be minimal, between 5–26 September 2014. *In situ* SeaPHOX loggers with SeaFET pH sensors were used to measure pH and DO hourly at these sites;

(3) In a moderately exposed site at 12.5 m (Fortescue Bay), most representative of *E. radiata* beds on the east coast of Tasmania and where pH change is likely to be intermediate between the situations described in 1) and 2), between 20-22 May 2014. An ENVCO pHTempion combined pH and temperature logger was used to measure pH at 5 min intervals as we did not have access to the primary sensors (the seaPHOX) at this time;

(4) Within sealed bags containing either seawater only, or seawater and an adult sporophyte of *E. radiata*, to determine the extent to which *E. radiata* metabolism can change pH in the field, and to separate the changes caused from photosynthesis and respiration of phytoplankton from that caused by *E. radiata*, at Darlington, on each of 18, 19 and 21 April 2014 using the pH meter, probe and DO probe described in 1); and

(5) Within the shallow (7 m) exposed *E. radiata* bed (described in 2) above) and on the adjacent soft-sediment substratum (Fortescue Bay, 20 m depth), to determine whether pH changes are localised within beds or evident over larger spatial scales, using a Niskin sampler and the pH meter, probe and DO probe described in 1) and 4), between 06:45 and 16:45 on 27 November 2014.

These five sampling procedures were used to capture a range of examples of pH change that could occur in *E. radiata* beds. Electrodes were calibrated initially using pH 7 and pH 9 NBS buffers on site, then pH on the total scale (pHT) was calculated afterwards using Tris and amp buffers at 14 °C. All pH measurements are given on the total scale, unless otherwise noted. The DO probe was calibrated by measuring the oxygen concentration of seawater that had been bubbled with air for 10 min (100% saturation) and by measuring the DO content of seawater that had been bubbled with N_2_ gas for 5 min (0%) at 14 °C. These methods of calibration were used for all measurements in the study, with the exception of the seaPHOX’s which were calibrated by taking DIC and A_T_ samples of seawater at deployment, a mid-point and collection. See supplementary methods (SI 1) for a full description and rationale of the methods of water sampling and pH measurement.

### Laboratory growth experiment 1: ambient seawater pH

Approximately 50 juvenile *Ecklonia radiata* individuals of blade length 40–100 mm were collected from Fortescue Bay (43.123079° S, 147.974848° E) on August 22^nd^ 2014 using SCUBA. Individuals were collected at 8–12 m, placed into black plastic bags containing seawater, and on the surface placed in an insulated container filled with seawater for transport (2 h) to the laboratory. Individuals were acclimated to laboratory conditions in 0.5 μm filtered (Whatman^®^ GF ⁄C filters; GE Healthcare UK Limited, Little Chalfont, UK) and UV sterilised (Emperor Aquatics Smart HO UV steriliser, 025050-2, 50 W lamp) seawater with constant aeration in darkness at 14 °C for 36 hours.

Intact sporelings (*n* = 24) were assigned randomly to one of 4 pH treatments (*n* = 6 for each treatment). We labelled treatments with names referring to the NBS scale to 1 decimal place, but measured pH on the total scale using Tris and amp buffers^48^ (see results for values). The treatments were: “pH 8.1”, “pH 7.8”, “pH 8.4”, and a “fluctuating pH” treatment which consisted of “pH 8.4” during the day and “pH 7.8” at night. All individuals were kept at 14 °C on a 12:12 light:dark cycle, with lights turned off at 20:00 and on at 08:00 in a temperature controlled room. Light levels during the day were 10 μmol photons m^−2^s^−1^ from day 1 to day 5 of the experiment to reduce stress associated with high light after collection (C.E. Cornwall, C.D. Hepburn and C.L. Hurd, unpublished data), and then raised to 28 μmol photons m^−2^s^−1^ from day 6 onwards, with the experiment conducted for a total of 21 days. One individual (replicate 5, constant pH 8.4) became necrotic early in the experiment and was discarded and excluded from analysis.

The pH of the experimental seawater collected from Bruny Island, Tasmania was modified either by bubbling with CO_2_ gas to reduce pH (chemically simulating biological release of CO_2_) or by bubbling with N_2_ gas to raise pH (simulating the biological uptake of CO_2_). These methods altered the concentration of DIC without altering A_T_, as is recommended^32^. Bubbling of N_2_ gas also removed dissolved O_2_ so subsequent re-equilibrium of O_2_ to 100% saturation was undertaken for the high pH treatments by bubbling O_2_ gas through the seawater. pH manipulations for each experimental treatment took place in the same container in a random order each day, and the same tubing was used for all gas delivery for every treatment to avoid pseudoreplication, following Fig. 3c from Cornwall and Hurd^51^. Modified water was placed in header tanks, which consisted of 5l low density polyethylene bags lined with a metallic film to keep pH constant for short term storage. Seawater then drained from the bags into the experimental tanks over time. The fluctuating pH treatment was achieved by changing bags over at 08:00, 11:00, 14:00, 17:00, and 20:00, with bags containing the appropriate seawater for the treatment, in other words using a step-wise approach^22^,^37^,^52^ and where pH was 8.4 in the day and 7.8 at night. Each individual was grown in a separate 650 ml culture tank with constant water motion (using magnetic stirrers) to break down the diffusion boundary layer. See^22^,^43^ for a description of the chambers. Trials were conducted to test the extent that juvenile *E. radiata* could alter pH in the culture tanks under the experimental light conditions (i.e. at 0 and 28 μmol photons m^−2^s^−1^), and revealed that tanks required exchange of seawater every 3 hours during the day and every 12 hours at night to ensure that pH remained within 0.05 units of the target.

### Growth experiment 2: OA conditions

Approximately 50 *E. radiata* sporelings were collected from the site at Fortescue Bay (as above) on August 20^th^ 2014. All experimental conditions and methods were the same as the growth experiment #1 described above, except that the target pH of each treatment was reduced by 0.3 pH_NBS_ units across all treatments to simulate conditions expected to occur in the future as a result of OA. Three individual sporophytes (two replicates subject to constant pH 7.5 and one replicate under constant pH 7.8) became necrotic and were discarded during the experiment.

### Biotic responses

Linear extension of the blade length (tip of blade to blade-stipe junction) of each sporophyte was calculated from photographs of individuals placed on a grid on days 1 and 21, using the software program ImageJ^53^. Relative growth rate was calculated relative to the initial size^54^.

Photosynthetic rates were determined on day 21 in each culture tank at experimental light levels at 11:00 after 3 hours in the light, using an Orion RDO probe (ORI087100MDW). Oxygen production was standardised to surface area of the thallus (in mm^2^) over an hour.

The performance of PSII (F_v_/F_m_) and maximum relative electron transport rates (rETR_max_) were measured to assess any changes in photo-physiology during the experiments. F_v_/F_m_ and rETR_max_ were measured using a Pulse Amplitude Modulation (PAM) chlorophyll fluorescence meter (Diving PAM, Walz, Germany) on day 21. F_v_/F_m_ measurements were made on *E. radiata* individuals that had been dark adapted for 15 minutes^55^. The diving PAM had a blue-light-emitting diode, and dampening and gain were set to 1 and 2 respectively. F_o_ was above 120 on all occasions.

Tissue samples from the meristem were taken at the end of the experiment on day 21 from each individual for determination of δ^13^C, C:N ratios, and RNA:DNA ratios. C:N was measured to indicate whether nutrient limitation occurred, and along with rETRmax and F_v_/F_m_, was used only as an indication of the physiological state of the algae. δ^13^C and C:N ratios were determined using the methods outlined in^39^ using a NA1500 elemental analyser coupled to a Thermo Scientific Delta V Plus via a Conflo IV. Combustion and reduction were achieved at 1020 °C and 650 °C respectively. Values were normalised to the VPDB scale via a 3 point calibration using certified reference material. Both precision and accuracy were ± 0.1‰ (1 SD). δ^13^C values were corrected to an absolute value (Δ^13^C) by determining the δ^13^C of the seawater for each treatment and subsequently using the formula: Δ^13^C  =  (δ^13^C_seawater_ − δ^13^C_tissue_)/(1 + δ^13^C_seawater_/1000)^43^. For analysis, one individual was removed from consideration of C:N ratios (treatment = constant pH 8.1) as this individual returned an extreme and unrealistic value substantially lower than all other replicates from the same treatment. See Supplementary Methods (SI 1) for details of RNA and DNA measurements.

Seawater samples were collected from each replicate at the beginning of a change in seawater pH and again after 3 hours. DIC concentrations of the water samples were measured using a DIC analyser (Apollo SciTech DIC analyser model AS-C3) with an inbuilt CO_2_ analyser (LI-COR LI-7000 CO_2_/H_2_O analyser). The CO_2_ analyser was calibrated with a certified reference material provided by Andrew Dickson, Scripps Institute for Oceanography, San Diego, USA^48^. A_T_ was calculated using the constants of Mehrbach^56^ and the refit by Dickson and Millero^57^.

### Statistical analysis

All statistical analyses were conducted using the statistical software *R* v. 3.1.1^58^. Correlations between pH and oxygen concentrations during field measurements were calculated using Pearson’s correlation coefficient. Two-way ANOVAs were used to assess whether there were differences between pH and oxygen concentration changes in bags placed in the field, with a fixed factor of ‘Treatment’ (2 levels: chambers containing *Ecklonia radiata* and control bags with no kelp) and a random factor ‘Day’ (3 levels: April 18^th^, 19^th^ and 21^st^). In the laboratory-based experiments, one-way ANOVAs were used to estimate whether there were significant differences between pH treatments for RGR, oxygen evolution, F_v_/F_m_, rETR_max_, C:N ratios, Δ^13^C, RNA:DNA ratios, and RNA and DNA content. When main effects in one-way ANOVAs were significant (at α = 0.05), Tukey’s Honest Significant Difference (THSD) post-hoc tests were used to determine the nature of differences between treatments. Data were checked for violations of ANOVA assumptions (normality and homoscedasticity), and passed all tests, except for Δ^13^C in the ambient pH experiment; 2 outliers were removed from the pH 8.1 treatment as they returned values that were substantially different to other replicates in the treatment.

## Additional Information

**How to cite this article**: Britton, D. *et al.* Ocean acidification reverses the positive effects of seawater pH fluctuations on growth and photosynthesis of the habitat-forming kelp, *Ecklonia radiata. Sci. Rep.* **6**, 26036; doi: 10.1038/srep26036 (2016).

## Supplementary Material

Supplementary Information

## Figures and Tables

**Table 1:** The range, mean, minimum and maximum pH values for all field measurements across the five deployments.

Site	Method	Season	Depth (m)	Habitat	Mean pH	Min. pH	Max. pH	pH range	Latitude andLongitude
Fortescue Bay	SeaPHOX, day/night	Spring	7.0	*E. radiata* bed	8.09	8.06	8.15	0.09	43.1234965° S147.977228° E
Fortescue Bay	SeaPHOX, day/night	Spring	25.0	*E. radiata* bed	8.09	8.06	8.11	0.05	43.12368°; S147.98136° E
Fortescue Bay	pHTempion, day/night	Autumn	12.5	*E. radiata* bed	8.05	8.01	8.09	0.08	43.123334°; S,147.975289° E
Darlington, Maria Island	pH electrode and bottle samples, day	Autumn	1.5	*E. radiata/Phyllospora comosa* bed	8.19	7.97	8.37	0.40	42.577494°; S,148.062957° E
Fortescue Bay	pH electrode and Niskin samples, day	Spring	7.0	*E. radiata* bed	8.16	8.13	8.19	0.06	43.1234965°; S147.977228° E
Fortescue Bay	pH electrode and Niskin samples, day	Spring	20.0	Soft sediment benthos	8.158	8.15	8.16	0.01	43.128708°; S,147.976640° E

All pH values are on the total scale.

**Table 2:** Analysis of variance (ANOVA) table displaying *F*-values, *P*-values, and Tukey Honestly Significantly Different (THSD) differences between treatments for all measured responses during the ambient and ocean acidification (OA) experiments.

Response	Ambient experiment			OA experiment		
	*F*-value	*P*-value	THSD	*F*-value	*P*-value	THSD
Blade length RGR	**5.434**	**0.007**	F > 8.4 = 8.1 = 7.8	2.356	0.100	
Photosynthetic rates	**14.72**	**<0.001**	F > 8.1 = 7.8	**3.840**	**<0.001**	7.8 > F
Δ^13^C	**4.385**	**0.020**	F < 8.1 = 7.8; 8.4 < 7.8	**4.782**	**0.014**	F < 7.5
*r*ETR_max_	1.435	0.264		2.680	0.080	
Fv/Fm	**4.551**	**0.015**	8.4 > 7.8	**5.500**	**0.008**	F < 8.1 = 7.8
RNA:DNA	**23.170**	**<0.001**	F = 8.4 > 8.1 = 7.5	0.950	0.438	
Total RNA	**9.530**	**<0.002**	F = 8.4 > 8.1 = 7.5	0.530	0.669	
C:N	**8.23**	**0.001**	F = 8.4 = 8.1 < 7.8	1.150	0.358	

Degrees of Freedom = 3 for Treatments and 21 to 17 for residuals. α = 0.05. For THSD, F = Fluctuating treatment, all other treatments named by their mean pH_NBS_.
