# Supplementary material for: Ocean acidification reverses the positive effects of seawater pH fluctuations on growth and photosynthesis of the habitat-forming kelp, Ecklonia radiata
Source: Sci Rep. 2016 May 27;6:26036. doi: 10.1038/srep26036 (PMC4882519; doi:10.1038/srep26036)
Supplement: Supplementary Information [file srep26036-s1.pdf]

Supplementary information for:

Ocean acidification reverses the positive effects of seawater pH fluctuations on growth and photosynthesis of the habitat-forming kelp, *Ecklonia radiata*

Damon Britton[1,*], Christopher E. Cornwall[1,2], Andrew T. Revill[3], Catriona L. Hurd[1], and Craig R. Johnson[1]

[1]Institute for Marine and Antarctic Studies, University of Tasmania, Hobart, TAS 7001, Australia

[2]School of Earth and Environment, Oceans Institue & ARC Centre of Excellence for Coral Reef Studies, University of Western Australia, Crawley, WA 6009, Australia

[3]CSIRO Oceans and Atmosphere, Hobart, TAS, Australia

[*]Corresponding author: Damon.Britton@outlook.com

Supplementary Figures:

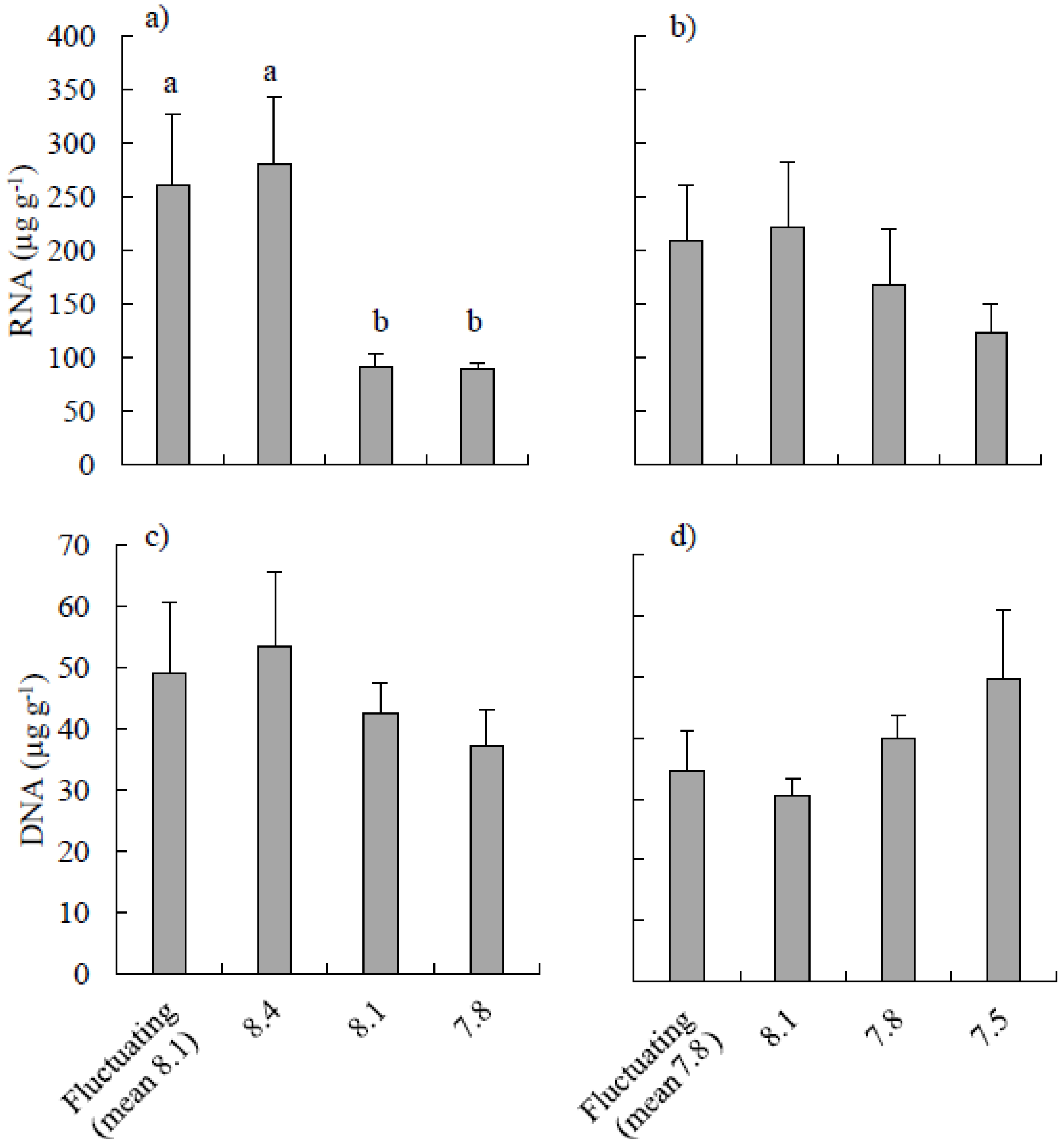

Figure S1:, content of a) RNA and c) DNA of juvenile *Ecklonia radiata*, measured after 21 days grown under ambient pH treatments: fluctuating pH (pH_{NBS} 8.4 during the day, 7.8 at night), pH_{NBS} 8.4, 8.1 and 7.8; b) RNA, and d) DNA under OA conditions: i.e. the same treatments as in (a and c) but with pH_{NBS} reduced by 0.3 units in each treatment. Data displayed as mean ± standard error, $n = 4 – 6$. Bars with different letters within panels are

significantly different, as revealed by Tukey's Honestly Significant Difference tests (α = 0.05).

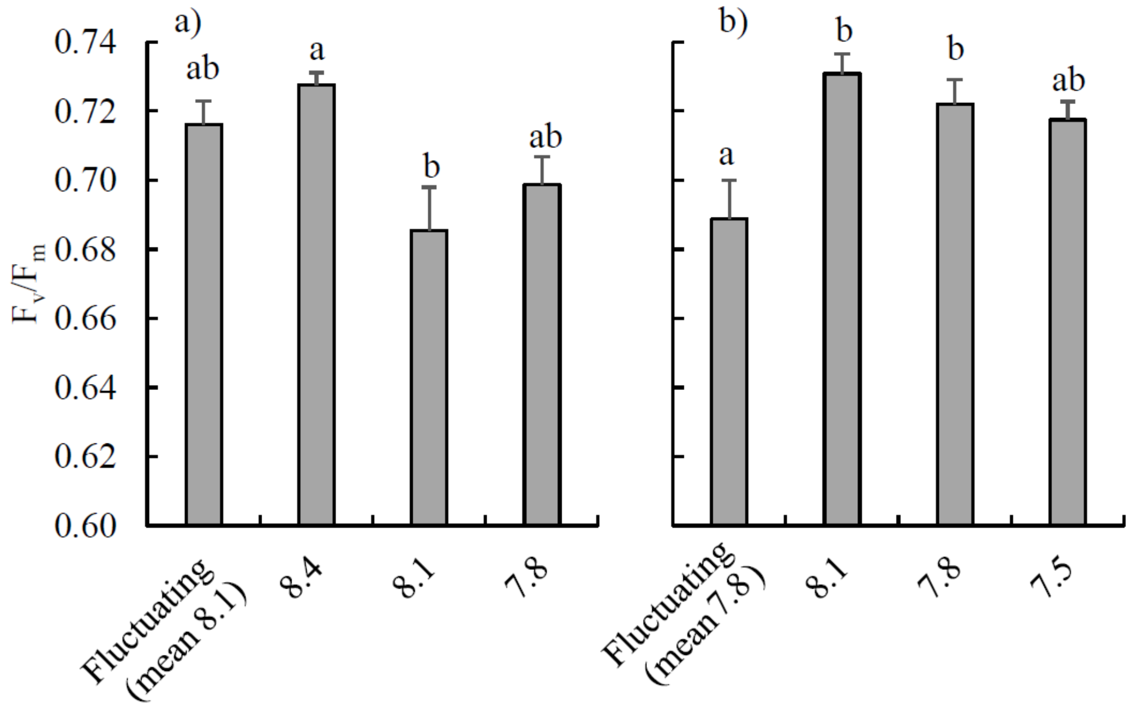

Figure S2: Dark adapted (15 min) F_v/F_m of juvenile *Ecklonia radiata* after 21 days, measured in the experimental tanks under a) ambient pH conditions: Fluctuating (pH_{NBS} 8.4 during the day, 7.8 at night), pH_{NBS} 8.4, 8.1 and 7.8; b) OA conditions: the same treatments as in (a) but with pH_{NBS} reduced by 0.3 units in each treatment. Data displayed as mean ± standard error, $n = 4 – 6$. Bars not sharing the same letter are significantly different within panels, as revealed by Tukey's Honestly Significant Difference tests ($\alpha = 0.05$).

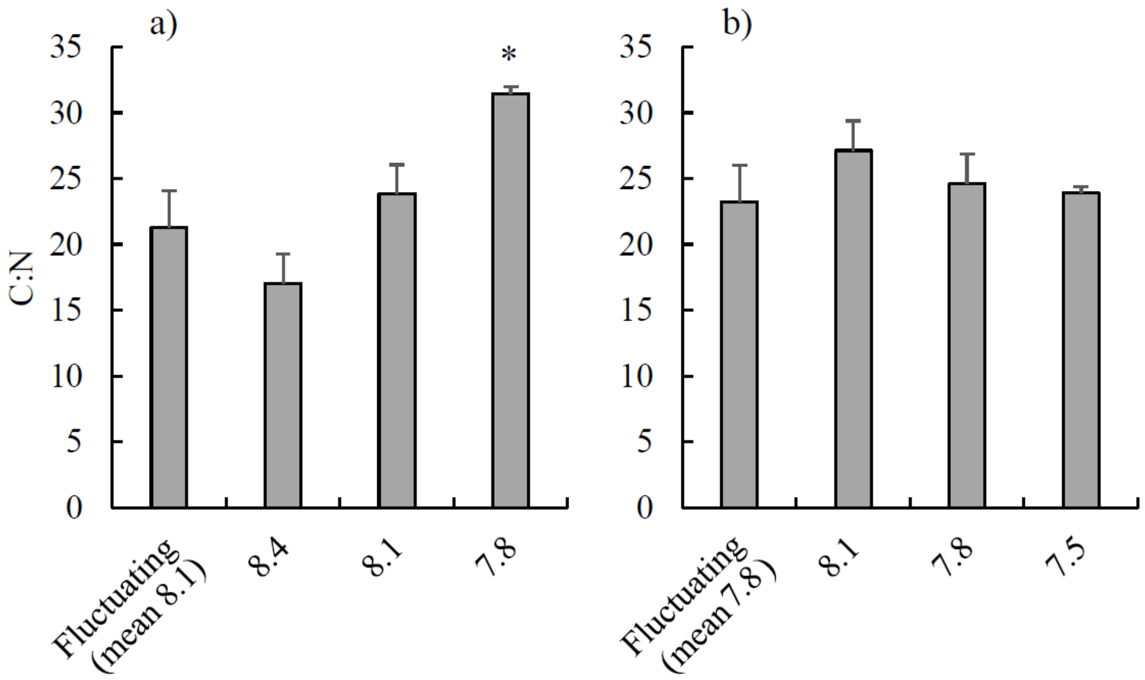

Figure S3: Carbon to nitrogen ratios of juvenile *Ecklonia radiata*, measured after grown 21 days under a) ambient pH conditions: Fluctuating (pH_{NBS} 8.4 during the day, 7.8 at night), pH_{NBS} 8.4, 8.1 and 7.8; b) OA conditions: the same treatments as in (a) but with pH_{NBS} reduced by 0.3 units in each treatment. Data displayed as mean ± standard error, $n = 4 – 6$. * denotes significantly different treatment, revealed by Tukey's Honestly Significant Difference tests ($\alpha = 0.05$).

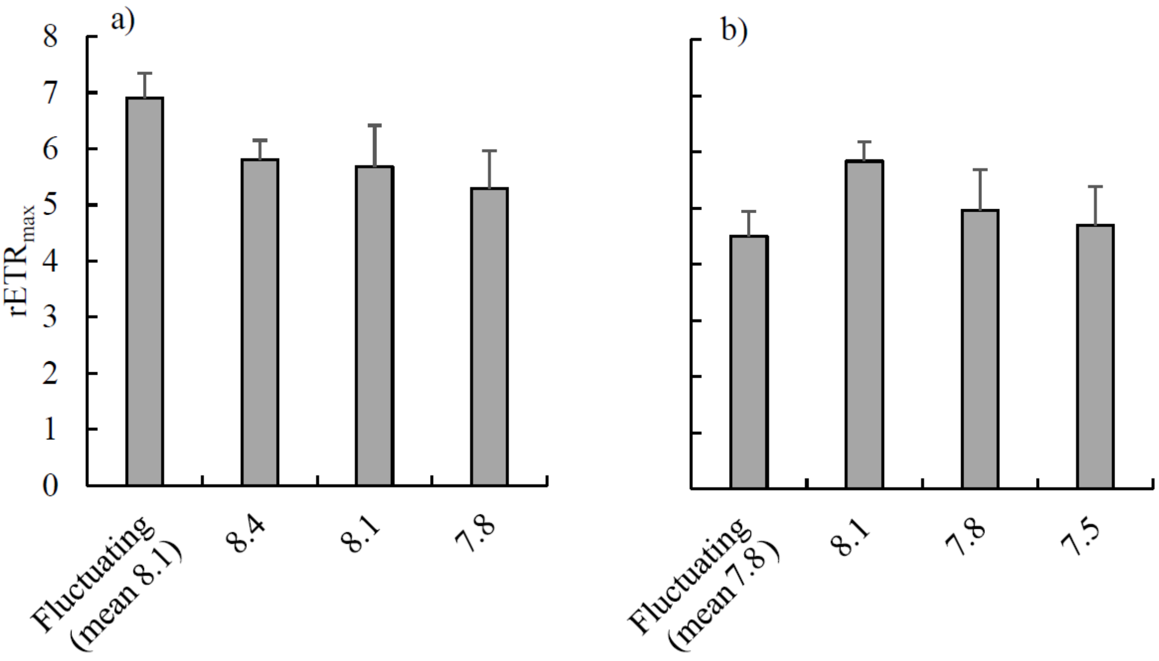

Figure S4: Maximum relative electron transport rates ($r\mathrm{ETR}_{max}$) of juvenile *Ecklonia radiata* grown for 21 days under a) ambient pH conditions: Fluctuating ($\mathrm{pH}_{\mathrm{NBS}}$ 8.4 during the day, 7.8 at night), $\mathrm{pH}_{\mathrm{NBS}}$ 8.4, 8.1 and 7.8; b) OA conditions: the same treatments as in (a) but with $\mathrm{pH}_{\mathrm{NBS}}$ reduced by 0.3 units in each treatment. Data displayed as mean ± standard error, $n = 4 – 6$.

Supplementary Tables:

Table S1. Measured mean daily seawater carbonate chemistry (pH_T and dissolved inorganic carbon [DIC] ± standard error), calculated total alkalinity (A_T) of experimental tanks housing juvenile kelp *Ecklonia radiata* over 21 days, and salinity of source seawater. pH in "Treatment" column indicate treatment labels, not measured values.

Treatment	pH_T	DIC (µmol kg^{-1})	A_T (µmol kg^{-1})
Fluctuating (ambient)	8.35 ± 0.01	1960 ± 14	2298
8.4	8.36 ± 0.01	1975 ± 40	2322
8.1	8.09 ± 0.01	2094 ± 15	2288
7.8	7.78 ± 0.01	2217 ± 18	2270
Fluctuating (ocean acidification)	8.06 ± 0.01	2185 ± 7	2368
8.1	8.11 ± 0.01	2183 ± 9	2391
7.8	7.80 ± 0.01	2248 ± 6	2329
7.5	7.54 ± 0.01	2323 ± 4	2321

Supplementary methods:

Field measurements

Sheltered and shallow *Ecklonia radiata* bed: Bottle samples (100 ml plastic sealed bottles) of seawater were collected *in situ* from within a sheltered and shallow (1.5m) mixed *Ecklonia radiata* and *Phyllospora comosa* bed at Darlington, Maria Island, Tasmania (42.577494° S, 148.062957° E). Bottle samples were used at that site because the *E. radiata* bed there was located in the surge zone, prohibiting the use of the sensors. Samples were collected at regular intervals between 07:30 and 17:30 on each day between April 19-21 2014. Four replicate bottles were collected at each sampling time. pH and oxygen concentration of the seawater was measured immediately after collection using a pH meter (Thermo Scientific Orion Star A216 pH/RDO/DO meter), pH electrode (Thermo Scientific Orion 8107 BNUMD Ross Ultra pH/ATC Triode) and dissolved oxygen (DO) probe (Thermo Scientific Orion 087100MD Field RDO probe). Electrodes were calibrated initially using pH 7 and pH 9 NBS buffers on site, then pH on the total scale (pH_T) was calculated afterwards on the total scale using Tris and amp buffers at 14°C. All pH measurements will be referred to on the total scale, unless otherwise noted. The DO probe was calibrated by measuring the oxygen concentration of seawater that had been bubbled with air for 10 min (100% saturation) and by measuring the DO content of seawater that had been bubbled with N_2 gas for 5 min (0%) at 14°C.

Exposed shallow and deep *E. radiata* beds (Fortescue Bay): Two SeapHOX loggers with SeaFET pH sensors were deployed from 5 – 26 September 2014 at the mouth of Fortescue Bay, one at 25 m depth (43.12368° S 147.98136° E) and the other at 7 m depth (43.1234965° S 147.977228° E). They recorded pH and oxygen concentration at half hourly intervals. The SeaPHOX deployment locations were slightly more exposed than the pHTempion deployments. 200 ml bottle samples for dissolved inorganic carbon (DIC) and

Total Alkalinity (A_T) were taken during deployment, towards the end of deployment (September 21st 2014) and during retrieval. All bottle samples were poisoned with mercuric chloride immediately following collection. DIC concentrations of bottle samples were determined using a SOMMA DIC analyser (University of Rhode Island) and Total Alkalinity (A_T) was determined using a Metrohm 904 alkalinity titrator. DIC and A_T of bottle samples were used to calibrate the SeaPHOX loggers. The SeaPHOX deployed at 25 m had a hardware error between 7 and 17 September, resulting in no pH measurements between those time dates.

Moderately exposed, mid-depth, *E. radiata* bed (Fortescue Bay): An ENVCO pHTempion combined pH and temperature logger was deployed in an *E. radiata* bed at 12:00 on May 20th 2014 at Fortescue Bay, Tasmania (43.123334° S, 147.975289° E) at a depth of 12.5 m. The logger recorded pH values at 5 min intervals and was collected at 11:30 on May 22nd.

Ecklonia radiata versus control seawater (Darlington): To differentiate between pH change caused by the focal species (*E. radiata*) and that caused by phytoplankton, individual 50 g *E. radiata* sporophytes were incubated in *in situ* clear polyethylene bags at 1.5 m depth at the Maria Island site, Darlington. On each of 18th, 19th and 21st April, four replicate bags were deployed together with four control chambers containing seawater (and therefore phytoplankton) but without macroalgae, see [1] for a description. All bags were deployed using SCUBA at 07:30 and collected at 16:30, and water samples were removed with syringes by divers at deployment and retrieval. The pH and the oxygen concentration of the water samples were measured immediately following return of syringes to the surface by divers, as per above methods.

Spatial measurements in exposed shallow and deep *Ecklonia radiata* beds, and nearby soft substrate (Fortescue Bay): On the open coast at Fortescue Bay, Tasmania, water samples were taken with a Niskin bottle approximately every two hours between 06:45 and 16:45 on November 27th 2014, at three locations: within the *E. radiata* canopy at 7 m (43.1234965° S 147.977228° E) and in the middle of the bay over the adjacent soft sediment benthos (sample depth = 20 m, 43.128708° S, 147.976640° E). At each sampling time and location, one sample of seawater was taken, and 3 measurements of pH was taken from each water sample, using the methods outlined for bottle samples.

Measurements of RNA and DNA concentrations

Fresh tissue obtained for RNA:DNA ratio analysis was immediately submersed in 'RNA-later' solution (Ambion, Inc., Austin, TX, USA), refrigerated overnight and then frozen at -20 C° as per the manufacturers recommendations. Frozen samples were thawed and blotted dry and 1-5 mg samples (wet weight) were weighed and mixed with a drill pestle in a solution of urea, sodium dodecyl sulphate (SDS) and Proteinase K solution. Solutions were then incubated at 37 °C for 10 minutes to ensure stabilisation of nucleic acids, complete cell lysis and digestion of RNAses. The solutions were then placed on ice and following cooling, ammonium acetate was added to each solution. Each solution was then vortexed and centrifuged for 5 mins at 14,000 RCF to precipitate impurities. The resulting supernatant was decanted into a 1.5 ml tube and precipitants discarded. Total nucleic acid (tNA) was pelletised by adding isopropanol, gently inverting the tube 40 times and centrifuging the tubes for 10 mins at 14, 000 RCF. The resulting tNA pellet was washed twice in 75% ethanol solution and suspended in 200 µl molecular grade H_2O and divided into two 100 µl aliquots. DNase (New England Biolabs - M0303L) was added to one aliquot to digest DNA while RNAse (Sigma Aldrich - R6148) was added to the second to digest RNA. Total RNA and total DNA were pelletised using the methods as for tNA. Both RNA and DNA pellets were

washed twice with 75% ethanol. The RNA pellet was suspended in molecular grade H_2O (100 µl) and the DNA pellet was suspended in an EB buffer solution. Concentrations of RNA and DNA were determined using fluorescence assays performed by a Quibit assay probe and fluorometer. RNA and DNA concentrations were expressed as total RNA and DNA content (µg.g wet weight^{-1}). The total concentrations were used to calculate RNA:DNA ratios. One individual (replicate 1, constant pH 8.1) displaying an RNA:DNA ratio ~1 order of magnitude higher than all others in that treatment was removed from the analysis under the suspicion that tissue not grown in the experiment was sampled for this replicate. A second individual (replicate 2, constant pH 7.8) returned an RNA concentration of zero and was excluded from any analysis since it was clear there were problems with the nucleic acid extraction.

References cited in the supplementary information:

1 Rodgers, K. L., Rees, T. A. V. & Shears, N. T. A novel system for measuring *in situ* rates of photosynthesis and respiration of kelp. *Marine Ecology Progress Series* **528**, 101-115 (2015).
